# Inhibitory Effect of Paquinimod on a Murine Model of Neutrophilic Asthma Induced by Ovalbumin with Complete Freund's Adjuvant

**DOI:** 10.1155/2021/8896108

**Published:** 2021-03-15

**Authors:** Jong-Uk Lee, Jong Sook Park, Ji Ae Jun, Min Kyung Kim, Hun Soo Chang, Dong Gyu Baek, Hyun Ji Song, Myung-Sin Kim, Choon-Sik Park

**Affiliations:** ^1^Department of Interdisciplinary Program in Biomedical Science Major, Soonchunhyang Graduate School, Bucheon, Republic of Korea; ^2^Division of Allergy and Respiratory Medicine, Department of Internal Medicine, Soonchunhyang University Bucheon Hospital, Bucheon, Republic of Korea; ^3^PulmoBioPark Co.,Ltd., Soonchunhyang University Bucheon Hospital, Bucheon, Republic of Korea; ^4^Department of Internal Medicine, Soonchunhyang University Gumi Hospital, Gumi, Gyeongsangbuk-do 39371, Republic of Korea

## Abstract

**Background:**

Quinoline-3-carboxamides have been used to treat autoimmune/inflammatory diseases in humans because they inhibit the functions of S100 calcium-binding protein A9 (S100A9), which participates in the development of neutrophilic inflammation in asthmatics and in an animal model of neutrophilic asthma. However, the therapeutic effects of these chemicals have not been evaluated in asthma. The purpose of this study was to evaluate the effect of paquinimod, one of the quinoline-3-carboxamides, on a murine model of neutrophilic asthma.

**Methods:**

Paquinimod was orally administered to 6-week-old C57BL/6 mice sensitized and challenged with ovalbumin (OVA)/complete Freund's adjuvant (CFA) and OVA. Lung inflammation and remodeling were evaluated using bronchoalveolar lavage (BAL) and histologic findings including goblet cell count. S100A9, caspase-1, IL-1*β*, MPO, IL-17, IFN-*γ*, and TNF-*α* were measured in lung lysates using western blotting.

**Results:**

Paquinimod restored the enhancement of airway resistance and the increases in numbers of neutrophils and macrophages of BAL fluids and those of goblet cells in OVA/CFA mice toward the levels of sham-treated mice in a dose-dependent manner (0.1, 1, 10, and 25 mg/kg/day, p.o.). Concomitantly, p20 activated caspase-1, IL-1*β*, IL-17, TNF-*α*, and IFN-*γ* levels were markedly attenuated.

**Conclusion:**

These data indicate that paquinimod effectively inhibits neutrophilic inflammation and remodeling in the murine model of neutrophilic asthma, possibly via downregulation of IL-17, IFN-*γ*, and IL-1*β*.

## 1. Background

Asthma is a heterogeneous disease consisting of various subtypes, which can be defined by their pathology, severity, etiology, physiological parameters, and response to treatment [1]. Cluster analysis of asthma cohorts has recently led to the identification of distinct clinical subphenotypes and different endotypes of asthma [2–4]. Airway inflammation is heterogeneously composed of eosinophilic, neutrophilic, mixed cellular, and paucigranulocytic types, each having distinct physiological and clinical characteristics [5, 6]. Specifically, about 40% of adult asthmatics have neutrophilic airway inflammation and 5%–10% of them have severe manifestations [5–7]. Although IL-8, TNF-*α*, interferon-*γ* (INF-*γ*), and IL-17 are assumed to be responsible for neutrophilic inflammation [8], therapies to protect against these molecules have not yet resulted in a good clinical response in severe asthmatic patients [9]. Thus, other molecular mediators should be regarded as therapeutic candidates for the treatment of neutrophilic inflammation. Recently, we showed that S100A9 initiates and amplifies neutrophilic inflammation in asthmatics and in animal models of asthma [10].

S100A9 is a small calcium-binding protein released by stressed cells undergoing necrosis that act as endogenous danger signals to accelerate and exacerbate the inflammatory response [11, 12]. This molecule induces signaling pathway of inflammatory responses such as chemotaxis [11, 12] and modulation of neutrophils and macrophages [13]. S100A9 protein binds to the proinflammatory receptors, receptor for advanced glycation end products (RAGE) and Toll-like receptor 4 (TLR4), both of which are involved in the pathogenesis of asthma [14, 15]. Transcripts of S100A9 are upregulated in peripheral blood mononuclear cells during asthma exacerbation [16]. Similarly, S100A9 is elevated in the sputum of neutrophilic inflammation in severe uncontrolled asthma [17]. Altered expression of the S100A9 protein is associated with lung diseases, such as cystic fibrosis, and other chronic inflammatory diseases, such as rheumatoid arthritis [18–20]. Paquinimod (ABR-215757, C_21_H_22_N_2_O_3_), one of the quinoline-3-carboxamide derivatives, targets the S100A9 protein by interrupting its binding to RAGE and TLR4. Additionally, paquinimod inhibits T-cell activation of NKT-II cells [21]. It has shown beneficial effects in several autoimmune/inflammatory disease models: paquinimod decreases pathology in experimental collagenase-induced osteoarthritis, systemic lupus erythematosus (SLE) patients [22] and in systemic sclerosis patients [23]. Thus, it was hypothesized that paquinimod would be effective in neutrophilic asthma. Recently, parts of the results have been published as the patent (PCT/KR2017/013799): paquinimod decreases the level of collagen, inflammation, and goblet cell in a murine model of asthma and that of pulmonary fibrosis. However, underlying mechanisms of paquinimod have not been defined up to date. The objectives of this study were to identify the effect of paquinimod on neutrophilic airway inflammation of experimental asthma using ovalbumin (OVA)/complete Freund's adjuvant- (CFA-) sensitized/challenged mice, which was generated as previously described [10]. We also evaluated the effects of paquinimod on the expression of Th1, Th17, and IL-1*β* because they are elevated and responsible for lung neutrophilia in this model [10].

## 2. Methods

### 2.1. Test Compounds and Formulations

Paquinimod (ABR-215757) (supplement Figure 1) was synthesized at J&H Co. (Seoul, Republic of Korea). The compound was dissolved in water, and the pH was adjusted to 7.5 and then dispensed in vials sufficient for one daily treatment, sealed, stored at 4°C, and used within 1 month of preparation.

### 2.2. Mice

In this study, forty-eight 6-week-old male C57BL/6 mice were purchased from ORIENTbio, Korea, and bred in a pathogen-free facility in the Laboratory Animal Research Center at Soonchunhyang University Bucheon Hospital. Animals were housed with ad libitum access to clean food and water with a 12 h light and dark cycle. All procedures were performed in accordance with the Declaration of Helsinki of the World Medical Association. The study protocol was approved by the Institutional Review Board (IRB)/Ethics Committee of Soonchunhyang University Hospital (SCHBC-animal-2014-012).

### 2.3. Paquinimod Treatment Protocol in the Neutrophilic Model of Murine Asthma

The neutrophil-dominant model was generated as we have previously described [10]. Briefly, mice (6 weeks of age, weighing 20–24 g) were sensitized via intraperitoneal injection of grade V chicken egg ovalbumin (OVA, 20 *µ*g; Sigma-Aldrich, St. Louis, MO) with complete Freund's adjuvant (CFA, 75 *µ*L; Sigma-Aldrich) emulsified in endotoxin-free phosphate-buffered saline (PBS) on days 0, 7, and 14. On days 21 and 22, all mice received intranasal challenges with 0.1% grade III OVA in 50 *µ*L saline. Control mice were administered saline instead. All procedures were performed in a sterile environment. Paquinimod (0.1, 1, 10, and 25 mg/kg/day) was administered via the drinking water from day 7 to day 23. Eight animals were used in each control and experimental group. All of the animals were alive during the study period. Mice were sacrificed 23 days after 20 *µ*g OVA with 75 *µ*L CFA intraperitoneal instillation, and one lung was taken for histology and one was homogenised for protein quantification, and the analysis of airway inflammation was conducted using bronchoalveolar lavage (BAL) cell analysis as described in supplement Figure 2. These mice were euthanized by cutting the inferior vena cava.

### 2.4. Measurement of Airway Resistance, BAL, and Histological Analysis

After being anesthetized with ketamine-xylazine, mice were tracheostomized and mechanically ventilated using the flexiVent system (Scireq, Montreal, Canada) 24 h later after the final OVA challenge as described in [24]. Airway resistance was measured using PBS and increasing doses of methacholine (0, 20, and 100 mg/mL; Sigma-Aldrich) and presented as the area under the curve (AUC) of the methacholine challenge. Immediately after the airway resistance was measured, BAL was performed, and lung tissues were processed for histology and protein measurements as described previously [25, 26]. Briefly, BAL was performed by four× instillation of 1 mL PBS and gentle retrieval. Cell numbers were measured using a hemocytometer, and differential cell counts were performed on slides prepared by cytocentrifugation and Diff-Quick staining (Scientific Products, Gibbstown, NJ), followed by counting 500 cells from each animal. BAL fluid was then centrifuged, and the supernatants were stored at −80°C.

A portion of the lung was fixed in 4% buffered paraformaldehyde and embedded in paraffin. The tissue was cut into 4 *µ*m thick slices, deparaffinized, rehydrolyzed, and stained with hematoxylin and eosin (H&E) and periodic acid-Schiff (PAS). Goblet cells were counted as the PAS-positive cells in the total number of bronchial epithelial cells from images captured at a magnification of 400× using a light microscope with ImageJ software (http://rsb.info.nih.gov/ij/) [27].

### 2.5. Western Blot

Western blot analysis was performed using mouse lung lysates as described previously [17]. Briefly, the extracted lung tissue samples were homogenised in a RIPA buffer containing 50 mM HC1 (pH 7.4), 50 mM NaCl, 0.1% SDS, 1% Triton X-100, 0.5 mM EDTA, and 100 mM phenylmethanesulfonyl fluoride in distilled water and centrifuged at 14,000 rpm for 30 min at 4°C. Lung lysate proteins (15 *μ*g/well) were loaded and electrophoresed on 15% polyacrylamide gels and then transferred to nitrocellulose membranes at 80 V for 120 min. The membranes were blocked for 1 h at room temperature in 5% skim milk in 0.1% Tween-20 in TBS and incubated overnight at 4°C with an anti-polyclonal S100A9 antibody (1 : 1,000 dilution; Novus Biological, Littleton, CA), anti-polyclonal caspase-1 antibody (1 : 1,000 dilution; Adipogen, San Diego, CA), anti-IL-1*β* monoclonal antibody (1 : 1,000 dilution; Cell Signaling Technology, Danvers, MA), anti-IFN-*γ* monoclonal antibody (1 : 1,000 dilution; Santa Cruz Biotechnology, Dallas, TX), anti-IL-17 monoclonal antibody (1 : 1,000 dilution; R&D Systems, Minneapolis, MN USA), anti-TNF-*α* monoclonal antibody (1 : 1,000 dilution; Abcam), anti-MPO monoclonal antibody (1 : 1,000 dilution; Abcam), and anti-*β*-actin monoclonal antibody (1 : 5,000 dilution; Sigma-Aldrich). The membranes were then incubated for 1 h at room temperature with a horseradish peroxidase-conjugated secondary antibody (1 : 5,000 dilution; Thermo, Fremont, CA). The target protein was detected by enhanced chemiluminescence solution (Amersham Pharmacia Biotech, Little Chalfont, UK) using X-ray film.

### 2.6. Statistical Analyses

The data were analyzed using statistical software package SPSS ver. 20.0. Comparisons of nonparametric variables were performed using Kruskal–Wallis tests, and then post hoc analysis using Mann–Whitney *U* tests was performed. All of the data were expressed as means ± standard error of the mean. *P* values of less than 0.05 were considered to be significant.

## 3. Results

### 3.1. Inhibitory Effect of Paquinimod on a Murine Model of Neutrophilic Asthma

**Figure 1:**
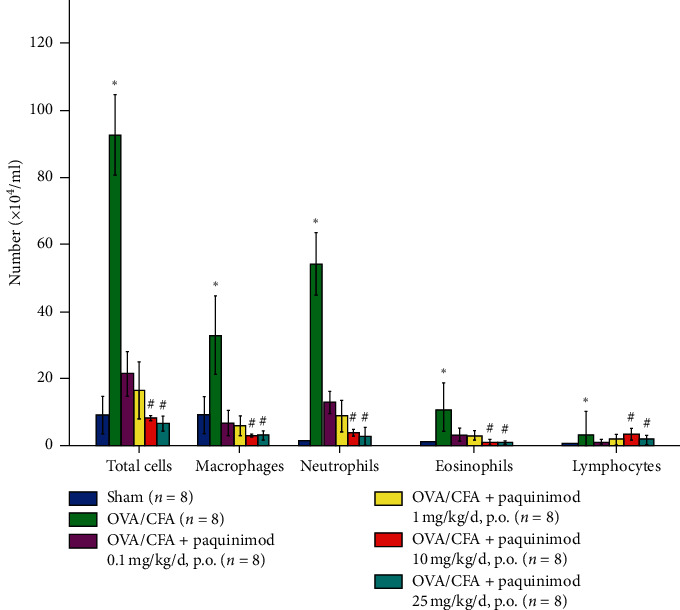
Effect of paquinimod on inflammatory cell number of CFA/OVA-treated mice. The numbers of BAL cells are shown as the mean ± SEM (× 10^4^/mL). ^*∗*^*P* < 0.05 compared with the sham group; ^#^*P* < 0.05 compared with the OVA/CFA group. The *P* value was obtained using the Kruskal–Wallis test and the post hoc Mann–Whitney *U* test.

Several dosages of paquinimod (0.1, 1, 10, and 25 mg/kg/day) were administered to 6-week-old C57BL/6 mice via the drinking water from day 7 to day 23 (Supplement Figure 2), and analysis of airway inflammation was conducted using BAL cell analysis and histological analysis. Paquinimod dose-dependently attenuated the increased number of inflammatory cells in the BAL fluid of CFA/OVA-sensitized/stimulated mice; 10 and 25 mg/kg/day of paquinimod significantly decreased the number of neutrophils, macrophages, eosinophils, and total cells (*P* < 0.05), whereas low doses (0.1 and 1 mg/kg/day) showed a decreasing effect (Figure 1).

**Figure 2:**
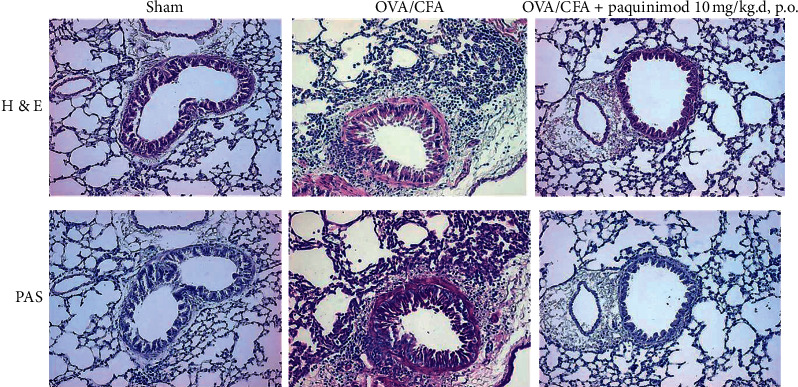
A representation picture of hematoxylin and eosin (H&E) and periodic acid-Schiff (PAS) staining of lung tissue. Lung sections were obtained from the sham- and OVA/CFA-treated mice with or without paquinimod treatment of 10 mg/kg/day, p.o. Tissue sections were stained with PAS to determine the presence of goblet cells (microscopy image (x200)).

**Figure 3:**
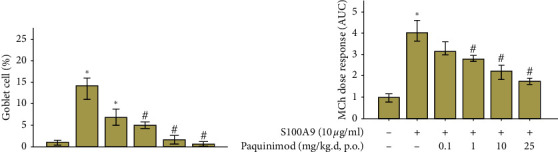
Effect of paquinimod on goblet cell count and lung resistance of OVA/CFA-treated mice. (a) Percentage of goblet cells in bronchial epithelium; periodic acid-Schiff (PAS)-positive cells and total epithelial cells were counted in the epithelium and the percentage of PAS-positive cells was calculated (*n* = 8 in each group). (b) Lung resistance of sham-treated or OVA/CFA-treated mice and OVA/CFA plus paquinimod (0.1 mg/kg/day to 25 mg/kg/day, p.o.) after challenges with increasing concentrations of methacholine (*n* = 8 in each group). ^*∗*^*P* < 0.05 compared with the sham group; ^#^*P* < 0.05 compared with the OVA/CFA group. The *P* value was obtained using the Mann–Whitney *U* test.

On H&E and PAS staining, numerous inflammatory cells were infiltrated around peribronchial and alveolar areas in the CFA/OVA-sensitized/stimulated mice with a concomitant increase of goblet cells (Figure 2). Treatment with paquinimod significantly and dose-dependently attenuated the increased numbers of inflammatory and goblet cells toward the numbers found in the sham-treated model (Figures 2 and 3(a)). Lung resistance measured using the flexiVent was significantly increased in CFA/OVA-sensitized/challenged mice, and paquinimod treatment attenuated the enhanced lung resistance in a dose-dependent manner (Figure 3(b)).

### 3.2. Effect of Paquinimod on S100A9 and Cytokines of the CFA/OVA Mice

**Figure 4:**
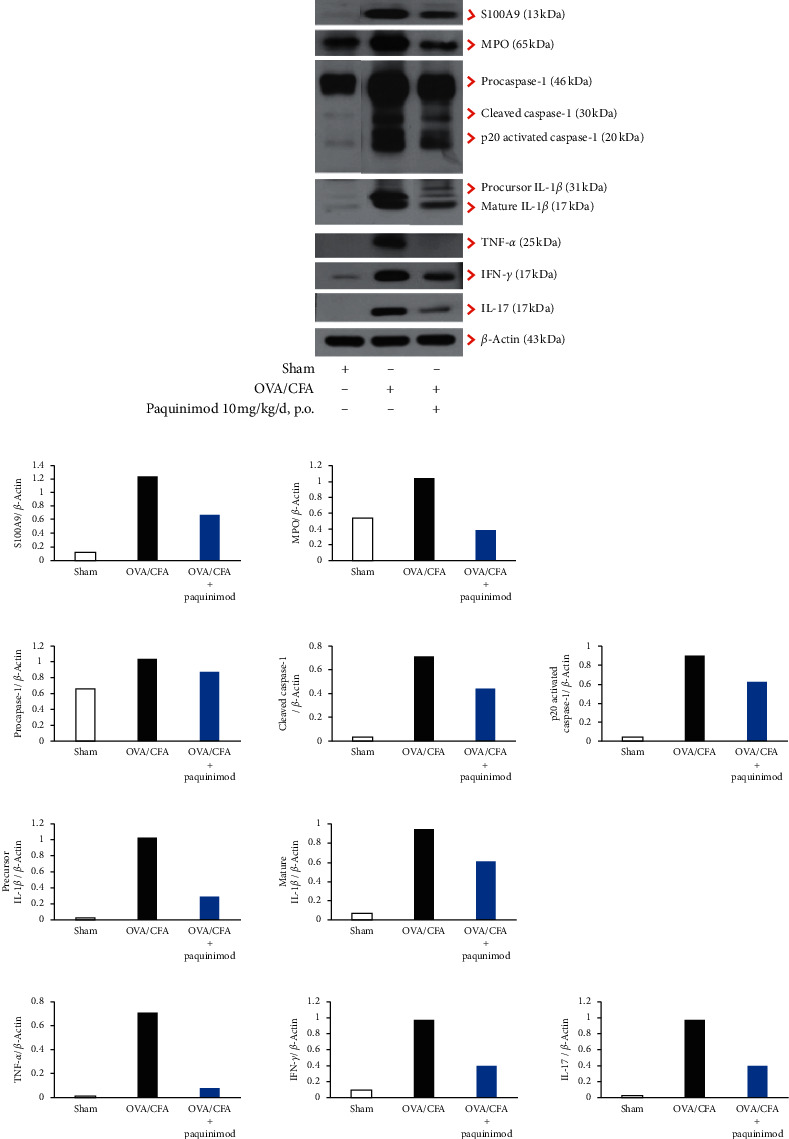
Western blot of inflammasomes, S100A9, and cytokines in mice lung lysates. (a) A representative figure of western blot of S100A9 (13 kDa), procaspase-1 (46 kDa), cleaved caspase-1 (30 kDa), p20 activated caspase-1 (20 kDa), precursor IL-1 *β* (31 kDa), mature form IL-1*β* (17 kDa), MPO (65 kDa), IL-17 (18 kDa), IFN-*γ* (17 kDa), and TNF-*α* (25 kDa) performed on the pooled lung lysates from sham-treated (*n* = 3), CFA/OVA-treated mice (*n* = 3), and CFA/OVA plus paquinimod (10 mg/kg/d, p.o.) (*n* = 3) (b) and their densitometry of the western blot band intensity normalized to that of *β*-actin.

Western blotting revealed that the lung tissues of CFA/OVA-sensitized and CFA/OVA-stimulated mice showed increases of S100A9, caspase-1, IL-1*β*, MPO, IL-17, IFN-*γ*, and TNF-*α* proteins. Treatment with paquinimod (10 mg/kg/day, p.o.) reduced the level of S100A9 with concomitant decreases of caspase-1, IL-1*β*, MPO, IL-17, IFN-*γ*, and TNF-*α* (Figure 4).

## 4. Discussion

We previously reported the relationship of S100A9 to the development of neutrophil-dominant inflammation in the CFA/OVA-induced murine model of asthma [10]. In the present study, we showed that paquinimod, a chemical inhibitor of S100A9 functions, significantly abolished the CFA/OVA-induced neutrophil-dominant inflammation and airway remodeling of goblet cell hyperplasia. To the best of our knowledge, this is the first report for the therapeutic effect of paquinimod against animal model of neutrophilic asthma. The S100A9 protein binds to the proinflammatory receptors involving RAGE and TLR4 [13], both of which are involved in the pathogenesis of asthma [15]. The binding of S100A9 to TLR4 induces NF-*κ*B activation and cytokine secretion in myeloid cells [28, 29]. Accordingly, paquinimod may be effective by competitively inhibiting both types of binding in our study.

The first development of quinoline-3-carboxamide analogues was in 1980, when Active Biotech in Sweden identified roquinimex, which later became famous by the trade name, Linomide [30]. Thereafter, compounds within this class exhibited potent disease-inhibitory effects in experimental models of autoimmune diseases including type II collagen arthritis model in mice [31], diabetic mice [32], autoimmune pathologies of the central and peripheral nervous systems [33], and experimental autoimmune encephalomyelitis [34]. Based on these results, Active Biotech screened the chemical library of quinoline-3-carboxamide analogues to identify second-generation compounds lacking proinflammatory side effects.

Paquinimod (ABR-215757) belongs to the second generation of quinoline-3-carboxamide derivatives, a class of structurally related small molecular compounds with immunomodulatory properties [35]. In the present study, several dosages of paquinimod (0.1, 1, 10, and 25 mg/kg/day) were administered to 6-week-old C57BL/6 mice via the drinking water for 2 weeks, with dosages of >1 mg/kg/day showing good inhibitory effects on neutrophilic inflammation and the remodeling of the mice. The dose was determined using an in vitro study as follows: inhibitory activity of paquinimod on S100A9 was performed by measuring NF-*κ*B luciferase activity of the 293-hTLR-MD2-CD14 transfected cells (InvivoGen, San Diego, CA, USA). The cells were transfected in 12-well plates with 2 *μ*g of NF-kB reporter vector (Panomics, Santa Clara, CA, USA) and 1 *μ*g of pRL-TK vector (Promega, Madison, USA) as an internal control. On the following day, the cells were treated with 10 *μ*g of S100A9 protein with various concentrations of paquinimod (0, 100, 500 nM, and 1 *μ*M) for 8 hours (Supplement Figure 3), and then, the luciferase activities of the cell lysates were measured by luminometer. As the result, S100A9-induced NF-KB luciferase activities were significantly reduced by paquinimod in a dose-dependent manner and IC50% of paquinimod was estimated to be about 878 nM. The level of paquinimod was not measured in the blood of the mice, which is a limitation of the present study. However, a pharmacokinetic study involving the systemic exposure of MRL-lpr/lpr mice to 0.04 and 0.2 mg/kg/day of paquinimod in the drinking water showed levels of 0.03 *µ*moles/L and 0.17 *µ*moles/L, respectively [22]. Thus, the dose of 1 mg/kg/day in the present study may result in the level of 0.85 *µ*moles/L (around the IC50%). In addition, paquinimod (0.1, 1, 10, and 25 mg/kg/day) was administered via the drinking water from day 7 to day 23 as shown in Supplemental Figure 2. This dose is higher than the dosage used in MRL-lpr/lpr mice and in human studies; paquinimod doses of 1.5 mg/day were well tolerated in healthy volunteers, and clinical doses of 1 mg/day and higher are predicted to be effective in the treatment of SLE patient [22] and, more recently, in systemic sclerosis patients [23]. Additionally, we did not observe any mouse mortality in the present study.

The S100A family proteins mediate inflammatory responses by acting on a variety of immune and nonimmune cells, including monocytes, neutrophils, endothelial cells, keratinocytes, and epithelial cells, with subsequent production of proinflammatory cytokines [28, 36, 37]. Additionally, S100A9 directly induces neutrophil degranulation and chemotaxis [38] and leucocyte adhesion and endothelial transmigration [39] which may be the main mechanism for airway neutrophilia in asthma. Furthermore, S100A family proteins are related to airway remodeling; MUC5AC mRNA and protein expression are induced by S100A8, S100A9, and S100A12 via several different signaling pathways [40]. Thus, our data of paquinimod-induced decrease of goblet cell number may be in good agreement with the S100A family protein-induced expression of MUC5AC mRNA. Moreover, S100A9 promotes fibroblast proliferation and upregulates collagen type III, *α*-smooth muscle actin, and receptors for advanced glycation end product expressions [37]. Upregulation of S100A9 and RAGE contributes to the enhanced basal migratory motility of fibrocytes in asthmatics experiencing acute exacerbation and those with chronic airflow limitations [41]. Accordingly, paquinimod may be effective on the thickened airway of asthmatics, although peribronchial smooth muscle thickness was not measured in the present study.

Regarding the molecular mechanism, the lung tissues of CFA/OVA-sensitized and CFA/OVA-stimulated mice showed increases of S100A9, caspase-1, IL-1*β*, MPO, IL-17, IFN-*γ*, and TNF-*α* proteins on western blots as similarly reported [10], and treatment with paquinimod significantly reduced the level of S100A9, with concomitant decreases of these proteins. In our previous animal study, we reported that administration of S100A9 to airways induced activation of inflammasomes and the production of IFN-*γ* and IL-17 [10]. However, the inhibitory effect of paquinimod may be incomplete in terms of S100A9 inhibition; S100A9-knockout mice had a more severe disease than C57BL/6 controls in experimental autoimmune encephalomyelitis, but they still responded to treatment with paquinimod [29]. Interestingly, biological redundancy may occur in the S100A9-knockout mice, maybe from the S100 family proteins (e.g., S100A12) [42] which serve as ligands for RAGE and TLR4 [29].

Recently, paquinimod has been reported to modify NKT-II cells to reduce the priming of proinflammatory effector CD4 (+) T cells, CD115^+^ Ly6C^hi^ monocytes, and CD11b^+^ F4/80^+^ CD206^+^ macrophages [21], although we did not analyze the effects of paquinimod on these immune responses, which will be studied in the future. Another limitation of the present study is a lack of an additional murine model of neutrophilic inflammation. The OVA/lipopolysaccharide (LPS) model has been used as a good model for neutrophilic airway inflammation [43], and high doses of LPS with OVA induce increased numbers of neutrophils with Th1 and Th17 immune responses [44]. Because LPS directly stimulates TLR4, which is a target molecule of paquinimod, the OVA/CFA model was preferred to the LPS/OVA model in the present study. The other limitation was that the BAL fluids were collected from both lungs, which might alter the number of goblet cells.

## 5. Conclusion

In the present study, we evaluated the effect of paquinimod, one of the quinoline-3-carboxamides, on a murine model of neutrophilic asthma, which was sensitized and challenged with ovalbumin (OVA)/complete Freund's adjuvant and OVA. Paquinimod restored the enhancement of airway resistance and the increases in numbers of neutrophils and macrophages of BAL fluids and goblet cell number in OVA/CFA mice toward the levels of sham-treated mice in a dose-dependent manner. Concomitantly, p20 activated caspase-1, IL-1*β*, IL-17, and IFN-*γ* levels were markedly attenuated. These results prove that paquinimod is an effective inhibitor against the neutrophilic inflammation and remodeling in a murine model of asthma, possibly via downregulation of IL-1*β*, IL-17, and IFN-*γ*. Taken together, paquinimod may be candidate therapeutics useful for neutrophilic asthma.

## Data Availability

The data used to support the findings of this study are available from the corresponding author upon request.

## Abbreviations

S100A9: S100 calcium-binding protein A9
TNF: Tumor necrosis factor
IFN: Interferon
IL: Interleukin
LPS: Lipopolysaccharide
BAL: Bronchoalveolar lavage
CFA: Complete Freund's adjuvant
OVA: Ovalbumin
PBS: Phosphate-buffered saline
TBS: Tris-buffered saline.

## Supplementary Materials

Supplementary Materials Supplement Figure 1: chemical structure of paquinimod (source: PubChem). C21H22N2O3, MW = 350.42. Supplement Figure 2: schematic protocol of treatment with paquinimod in OVA/CFA-stimulated and sensitized C57BL/6. Lung resistance, bronchoalveolar lavage, and tissue processing were performed on day 23. IN: intranasal; *R*_L_ test: lung resistance. Lung resistance (*R*_L_: cmH_2_O/s/ml) was measured using the flexiVent with increasing concentrations of methacholine. Supplement Figure 3: effects of dose-dependent paquinimod on NF-*κ*B luciferase activity. Cells positive for 293-hTLR4A-MD2-CD13 were transfected with NF-*κ*B luciferase and pRL-Tk constructs, and luciferase activity was measured 8 h after treatment recombinant S100A9 10 *μ*g and paquinimod (0, 100, 500 nM, and 1 uM). Data are means ± SEM of six experiments; ^*∗*^*P* < 0.05, compared with PBS, ^#^*P* < 0.05, compared with S100A9. *P* value was obtained using Mann–Whitney *U* test.

## References

[1] Green R. H., Brightling C. E., Bradding P. (2007). The reclassification of asthma based on subphenotypes. *Current Opinion in Allergy & Clinical Immunology*.

[2] Haldar P., Pavord I. D., Shaw D. E. (2008). Cluster analysis and clinical asthma phenotypes. *American Journal of Respiratory and Critical Care Medicine*.

[3] Kim T.-B., Jang A.-S., Kwon H.-S. (2013). Identification of asthma clusters in two independent Korean adult asthma cohorts. *European Respiratory Journal*.

[4] Park S. Y., Chang Y. S., Yoon S. Y. (2013). Clinical significance of asthma clusters by longitudinal analysis in Korean asthma cohort. *PLoS One*.

[5] Wenzel S. E., Schwartz L. B., Langmack E. L. (1999). Evidence that severe asthma can be divided pathologically into two inflammatory subtypes with distinct physiologic and clinical characteristics. *American Journal of Respiratory and Critical Care Medicine*.

[6] Choi J.-S., Jang A. S., Park J. S. (2012). Role of neutrophils in persistent airway obstruction due to refractory asthma. *Respirology*.

[7] Moore W. C., Hastie A. T., Li X. (2014). Sputum neutrophil counts are associated with more severe asthma phenotypes using cluster analysis. *Journal of Allergy and Clinical Immunology*.

[8] Shannon J., Ernst P., Yamauchi Y. (2008). Differences in airway cytokine profile in severe asthma compared to moderate asthma. *Chest*.

[9] Wenzel S. E., Barnes P. J., Bleecker E. R. (2009). A randomized, double-blind, placebo-controlled study of tumor necrosis factor-*α* blockade in severe persistent asthma. *American Journal of Respiratory and Critical Care Medicine*.

[10] Lee T.-H., Chang H. S., Bae D.-J. (2017). Role of S100A9 in the development of neutrophilic inflammation in asthmatics and in a murine model. *Clinical Immunology*.

[11] Ryckman C., McColl S. R., Vandal K. (2003). Role of S100A8 and S100A9 in neutrophil recruitment in response to monosodium urate monohydrate crystals in the air-pouch model of acute gouty arthritis. *Arthritis & Rheumatism*.

[12] Ryckman C., Vandal K., Rouleau P., Talbot M., Tessier P. A. (2003). Proinflammatory activities of S100: proteins S100A8, S100A9, and S100A8/A9 induce neutrophil chemotaxis and adhesion. *The Journal of Immunology*.

[13] Cesaro A., Plante A., Page N. (2012). An inflammation loop orchestrated by S100A9 and calprotectin is critical for development of arthritis. *PLoS One*.

[14] Simpson J. L., Milne D. G., Gibson P. G. (2009). Neutrophilic asthma has different radiographic features to COPD and smokers. *Respiratory Medicine*.

[15] Di Candia L., Gomez E., Venereau E. (2017). HMGB1 is upregulated in the airways in asthma and potentiates airway smooth muscle contraction via TLR4. *Journal of Allergy and Clinical Immunology*.

[16] Aoki T., Matsumoto Y., Hirata K. (2009). Expression profiling of genes related to asthma exacerbations. *Clinical & Experimental Allergy*.

[17] Lee T.-H., Jang A.-S., Park J.-S. (2013). Elevation of S100 calcium binding protein A9 in sputum of neutrophilic inflammation in severe uncontrolled asthma. *Annals of Allergy, Asthma & Immunology*.

[18] Roth J., Wilke M., Grun L. (1992). Complex pattern of the myelo-monocytic differentiation antigens MRP8 and MRP14 during chronic airway inflammation. *Immunobiology*.

[19] Goebeler M., Roth J., Burwinkel F., Vollmer E., Böcker W., Sorg C. (1994). Expression and complex formation of S100-like proteins MRP8 and MRP14 by macrophages during renal allograft rejection. *Transplantation*.

[20] Brun J. G., Jonsson R., Haga H. J. (1994). Measurement of plasma calprotectin as an indicator of arthritis and disease activity in patients with inflammatory rheumatic diseases. *The Journal of Rheumatology*.

[21] Fransen Pettersson N., Julia N., Tine D. H. (2018). The immunomodulatory quinoline-3-carboxamide paquinimod reverses established fibrosis in a novel mouse model for liver fibrosis. *PLoS One*.

[22] Bengtsson A. A., Sturfelt G., Lood C. (2012). Pharmacokinetics, tolerability, and preliminary efficacy of paquinimod (ABR-215757), a new quinoline-3-carboxamide derivative: studies in lupus-prone mice and a multicenter, randomized, double-blind, placebo-controlled, repeat-dose, dose-ranging study in. *Arthritis & Rheumatism*.

[23] Hesselstrand R. D. J., Riemekasten G., Törngren M., Nyhlén H. C., Stenström M. (2014). An open-label study to evaluate biomarkers and safety in systemic sclerosis (SSc) patients treated with paquinimod (ABR-215757). *Annals of the Rheumatic Diseases*.

[24] Park S.-W., Lee E. H., Lee E.-J. (2013). Apolipoprotein A1 potentiates lipoxin A4 synthesis and recovery of allergen-induced disrupted tight junctions in the airway epithelium. *Clinical & Experimental Allergy*.

[25] Jang A.-S., Choi I.-S., Takizawa H. (2005). Additive effect of diesel exhaust particulates and ozone on airway hyperresponsiveness and inflammation in a mouse model of asthma. *Journal of Korean Medical Science*.

[26] Lee S.-H., Kim K.-H., Kim J.-M. (2011). Relationship between group-specific component protein and the development of asthma. *American Journal of Respiratory and Critical Care Medicine*.

[27] Zhu Y. (2015). Baseline goblet cell mucin secretion in the airways exceeds stimulated secretion over extended time periods, and is sensitive to shear stress and intracellular mucin stores. *PLoS One*.

[28] Riva M., Källberg E., Björk P. (2012). Induction of nuclear factor-*κ*B responses by the S100A9 protein is Toll-like receptor-4-dependent. *Immunology*.

[29] Björk P., Björk A., Vogl T. (2009). Identification of human S100A9 as a novel target for treatment of autoimmune disease via binding to quinoline-3-carboxamides. *PLoS Biology*.

[30] Gupta N., Al Ustwani O., Shen L., Pili R. (2014). Mechanism of action and clinical activity of tasquinimod in castrate-resistant prostate cancer. *OncoTargets and Therapy*.

[31] Bjork J., Kleinau S. (1989). Paradoxical effects of LS-2616 (Linomide) treatment in the type II collagen arthritis model in mice. *Agents Actions*.

[32] Gross D. J., Sidi H., Weiss L., Kalland T., Rosenmann E., Slavin S. (1994). Prevention of diabetes mellitus in non-obese diabetic mice by Linomide, a novel immunomodulating drug. *Diabetologia*.

[33] Hedlund G., Link H., Zhu J., Xiao B.-G. (2001). Effects of Linomide on immune cells and cytokines inhibit autoimmune pathologies of the central and peripheral nervous system. *International Immunopharmacology*.

[34] Brunmark C., Sparre B., Anna R. (2002). The new orally active immunoregulator laquinimod (ABR-215062) effectively inhibits development and relapses of experimental autoimmune encephalomyelitis. *Journal of Neuroimmunology*.

[35] Jönsson S., Andersson G., Fex T. (2004). Synthesis and biological evaluation of new 1, 2-Dihydro-4-hydroxy-2-oxo-3-quinolinecarboxamides for treatment of autoimmune disorders: structure−Activity relationship. *Journal of Medicinal Chemistry*.

[36] Vogl T., Tenbrock K., Ludwig S. (2007). Mrp8 and Mrp14 are endogenous activators of Toll-like receptor 4, promoting lethal, endotoxin-induced shock. *Nature Medicine*.

[37] Xu X., Chen H., Zhu X. (2013). S100A9 promotes human lung fibroblast cells activation through receptor for advanced glycation end-product-mediated extracellular-regulated kinase 1/2, mitogen-activated protein-kinase and nuclear factor-*κ*B-dependent pathways. *Clinical & Experimental Immunology*.

[38] Simard J.-C., Girard D., Tessier P. A. (2010). Induction of neutrophil degranulation by S100A9 via a MAPK-dependent mechanism. *Journal of Leukocyte Biology*.

[39] Viemann D., Strey A., Janning A. (2005). Myeloid-related proteins 8 and 14 induce a specific inflammatory response in human microvascular endothelial cells. *Blood*.

[40] Kang J. H., Hwang S. M., Chung I. Y. (2015). S100A8, S100A9 and S100A12 activate airway epithelial cells to produce MUC5AC via extracellular signal-regulated kinase and nuclear factor-kappaB pathways. *Immunology*.

[41] Wang C.-H., Punde T. H., Huang C.-D. (2015). Fibrocyte trafficking in patients with chronic obstructive asthma and during an acute asthma exacerbation. *Journal of Allergy and Clinical Immunology*.

[42] Foell D., Wittkowski H., Kessel C. (2013). Proinflammatory S100A12 can activate human monocytes via Toll-like receptor 4. *American Journal of Respiratory and Critical Care Medicine*.

[43] Kim Y.-K., Oh S.-Y., Jeon S. G. (2007). Airway exposure levels of lipopolysaccharide determine type 1 versus type 2 experimental asthma. *The Journal of Immunology*.

[44] Yu Q. L., Chen Z. (2018). Establishment of different experimental asthma models in mice. *Experimental and Therapeutic Medicine*.

