# Corrigendum to “Inhibitory Effect of Paquinimod on a Murine Model of Neutrophilic Asthma Induced by Ovalbumin with Complete Freund's Adjuvant”

**DOI:** 10.1155/2021/9795069

**Published:** 2021-06-04

**Authors:** Jong-Uk Lee, Jong Sook Park, Ji Ae Jun, Min Kyung Kim, Hun Soo Chang, Dong Gyu Baek, Hyun Ji Song, Myung-Sin Kim, Choon-Sik Park

**Affiliations:** ^1^Department of Interdisciplinary Program in Biomedical Science Major, Soonchunhyang Graduate School, Bucheon, Republic of Korea; ^2^Division of Allergy and Respiratory Medicine, Department of Internal Medicine, Soonchunhyang University Bucheon Hospital, Bucheon, Republic of Korea; ^3^PulmoBioPark Co.,Ltd., Soonchunhyang University Bucheon Hospital, Bucheon, Republic of Korea; ^4^Department of Internal Medicine, Soonchunhyang University Gumi Hospital, Gumi, Gyeongsangbuk-do 39371, Republic of Korea

In the article titled “Inhibitory Effect of Paquinimod on a Murine Model of Neutrophilic Asthma Induced by Ovalbumin with Complete Freund's Adjuvant” [1], due to an error during the preparation of the manuscript, the incorrect authors' contributions statement and funding statement were included in the published article. The correct acknowledgments section should read as follows.
