# Supplementary material for: Inhibitory Effect of Paquinimod on a Murine Model of Neutrophilic Asthma Induced by Ovalbumin with Complete Freund's Adjuvant
Source: Can Respir J. 2021 Mar 15;2021:8896108. doi: 10.1155/2021/8896108 (PMC7984926; doi:10.1155/2021/8896108)

**Supplemental Figure 1**

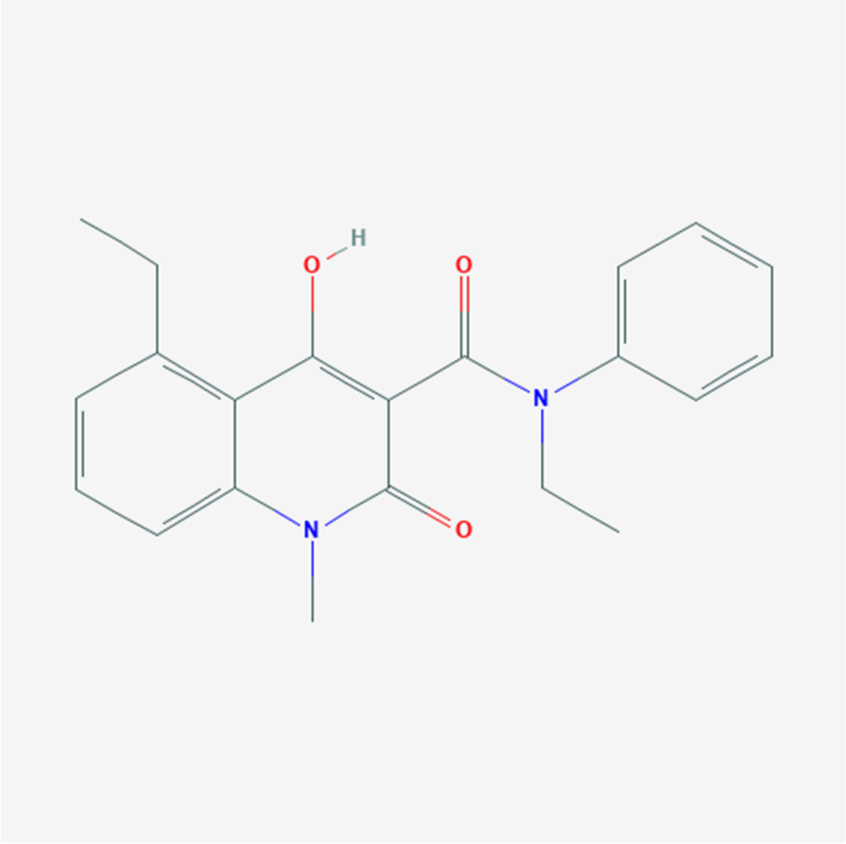

**Supplemental Figure 2**

-Neutrophilic asthma model using C57BL mice(OVA/CFA) and treatment with paquinimod

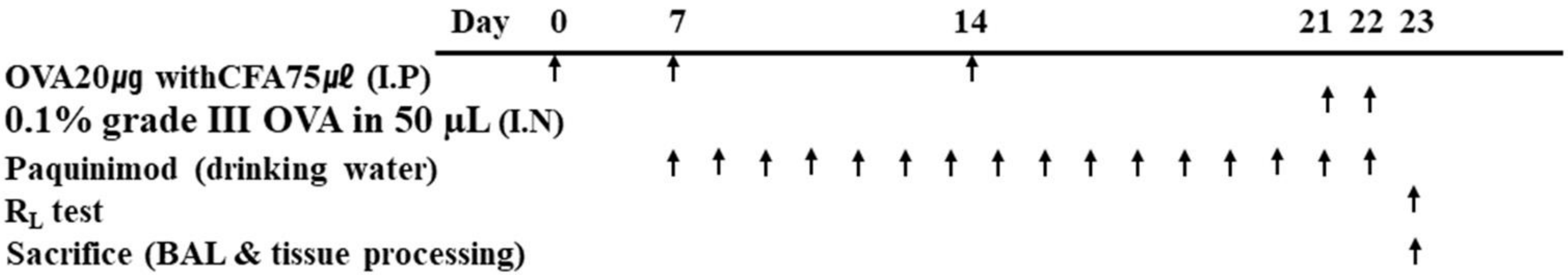

**Supplemental Figure 3**

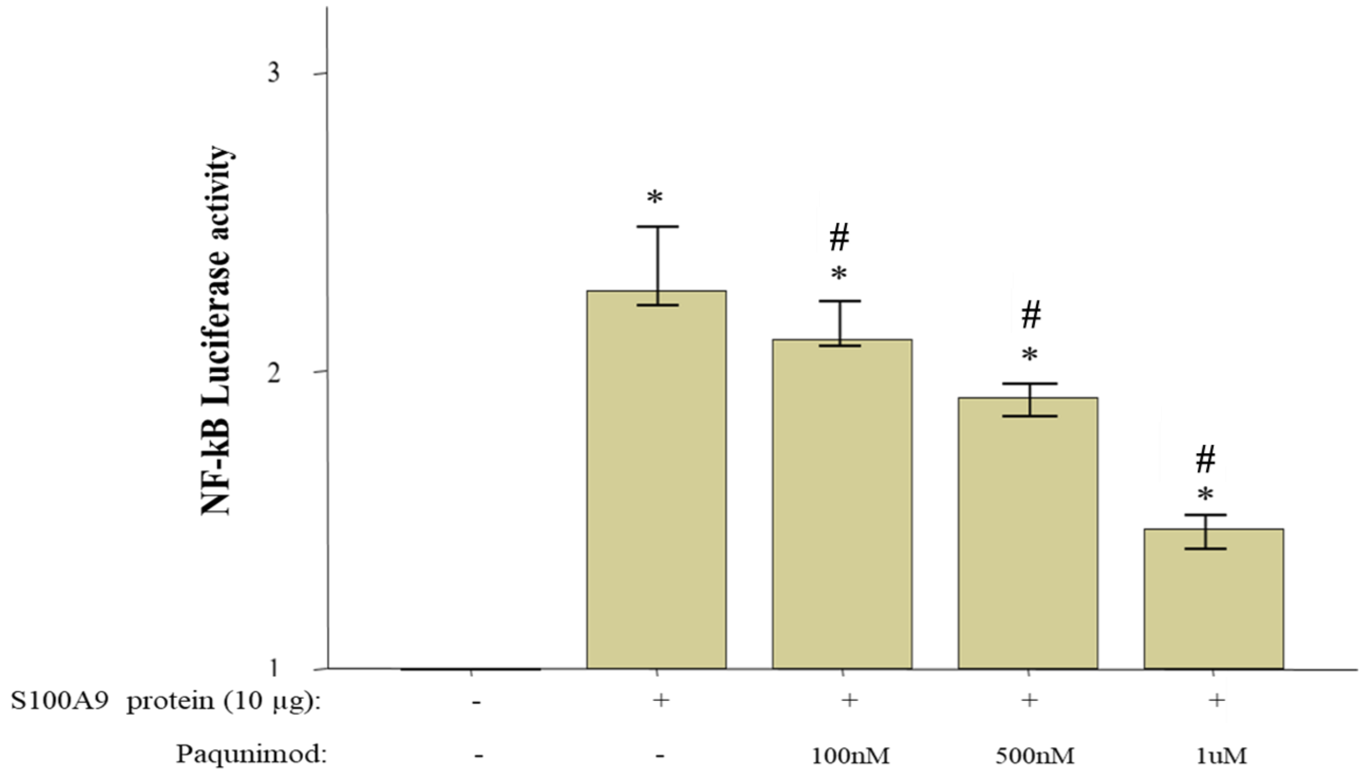

Supplement: Supplementary Materials — Supplement Figure 1: chemical structure of paquinimod (source: PubChem). C21H22N2O3, MW = 350.42. Supplement Figure 2: schematic protocol of treatment with paquinimod in OVA/CFA-stimulated and sensitized C57BL/6. Lung resistance, bronchoalveolar lavage, and tissue processing were performed on day 23. IN: intranasal; RL test: lung resistance. Lung resistance (RL: cmH2O/s/ml) was measured using the flexiVent with increasing concentrations of methacholine. Supplement Figure 3: effects of dose-dependent paquinimod on NF-κB luciferase activity. Cells positive for 293-hTLR4A-MD2-CD13 were transfected with NF-κB luciferase and pRL-Tk constructs, and luciferase activity was measured 8 h after treatment recombinant S100A9 10 μg and paquinimod (0, 100, 500 nM, and 1 uM). Data are means ± SEM of six experiments; ∗P < 0.05, compared with PBS, #P < 0.05, compared with S100A9. P value was obtained using Mann–Whitney U test. [file 8896108.f1.pdf]
